# Revolutionising Breast Surgery: A Comprehensive Review of Robotic Innovations in Breast Surgery and Reconstruction

**DOI:** 10.7759/cureus.52695

**Published:** 2024-01-21

**Authors:** Yashraj Jain, Ranjana Lanjewar, Raju K Shinde

**Affiliations:** 1 Department of General Surgery, Jawaharlal Nehru Medical College, Datta Meghe Institute of Higher Education and Research, Wardha, IND

**Keywords:** ethical considerations, minimally invasive procedures, surgical innovation, patient outcomes, precision surgery, robotic breast surgery

## Abstract

Robotic innovations in breast surgery have ushered in a new era of precision, safety, and patient-centred care. This comprehensive review explores the multifaceted realm of robotic breast surgery, from preoperative planning to postoperative outcomes, learning curves for surgeons, and the implications for healthcare policies. We examine the ethical considerations, cost-effectiveness, and future directions, including integrating artificial intelligence and telesurgery. Key findings reveal that robotic systems provide improved surgical precision, reduced complications, and enhanced patient satisfaction. Ethical concerns encompass informed consent, resource allocation, and equitable access. The future of breast surgery lies in continued research and development, ensuring that robotics becomes a standard of care accessible to all patients. This technology is reshaping breast surgery and offering new possibilities for minimally invasive, patient-centred care, ultimately redefining the standards of care in this critical field of medicine.

## Introduction and background

Breast surgery and reconstruction have been pivotal in treating and recovering breast cancer patients and individuals seeking aesthetic enhancements. These surgical procedures, which often encompass mastectomies, lumpectomies, nipple-sparing surgeries, and breast reconstructions, are integral to holistic care and emotional well-being for those affected by breast-related health challenges. Over the years, advancements in surgical techniques and technologies have continued to refine these procedures, enhancing their safety and efficacy [1,2].

Breast surgery encompasses a broad spectrum of procedures, with a common goal of treating breast cancer, addressing benign breast conditions, or pursuing aesthetic modifications. For breast cancer patients, surgical interventions, such as mastectomy and lumpectomy, are often necessary to remove tumours or affected tissue. Subsequent breast reconstruction can help restore the physical appearance and self-esteem of patients undergoing these procedures. Additionally, breast surgery extends to cosmetic procedures, which allow individuals to alter their breast size and shape for personal or aesthetic reasons [3]. In recent years, breast surgery and reconstruction have seen considerable advancements that aim to improve surgical precision and outcomes and prioritise the psychological and emotional aspects of the patient’s experience. These advancements have sparked a significant transformation in the field, with the introduction of robotics as a notable driving force [4].

The integration of robotics into breast surgery and reconstruction represents a remarkable leap forward in the realm of surgical techniques. Robotic innovations offer a unique set of advantages that have the potential to enhance the quality of care provided to patients undergoing breast-related surgical procedures [5]. Robotic systems provide surgeons with increased dexterity, precision, and three-dimensional (3D) visualisation, allowing them to perform complex procedures with greater accuracy and control. This, in turn, can lead to reduced postoperative complications, shorter recovery times, and improved cosmetic results. Moreover, the implementation of robotic technology in breast surgery has the potential to minimise the invasiveness of procedures, making them less traumatic and more patient-friendly [6].

This comprehensive review aims to delve into the remarkable transformation brought about by robotic innovations in breast surgery and reconstruction. It encompasses several vital objectives. First and foremost, it aims to provide an overview of breast surgery’s historical evolution, tracing robotic technology’s integration within this domain. This historical context sets the stage for discussing the current state of robotic systems employed in breast surgery, allowing for comparing different platforms and exploring their respective advantages and limitations.

## Review

Search methodology

The methodology employed in this comprehensive review involves a systematic approach to curate and analyse relevant literature on the integration of robotic innovations in breast surgery. A thorough search was conducted across PubMed, Scopus, and Web of Science databases using keywords such as “robotic breast surgery” and “breast reconstruction.” Inclusion criteria focused on articles contributing to the understanding of robotic technology’s impact on surgical techniques and patient outcomes, while exclusion criteria filtered out studies lacking peer review or not published in English.

Robotic systems in breast surgery

Overview of Various Robotic Surgical Systems

da Vinci surgical system (Intuitive Surgical, Inc., Sunnyvale, California, United States): The da Vinci system stands as one of the most renowned and widely adopted robotic platforms in the surgery world. Its exceptional dexterity, precision, and state-of-the-art 3D visualisation system distinguished it. These features make it particularly valuable in breast surgeries, especially those requiring minimally invasive techniques. Surgeons benefit from enhanced visualisation and precise control when performing intricate procedures in delicate breast tissue [7].

Mazor X Stealth Edition (Medtronic plc, Dublin, Ireland): Originally developed for spinal surgeries, the Mazor X Stealth Edition has been adapted for breast surgery applications. This system excels at merging preoperative planning with intraoperative navigation, equipping surgeons with the tools to make accurate and meticulous incisions and tissue removal. By seamlessly integrating planning and execution, it enhances the precision and safety of breast procedures [8].

Senhance® Surgical System (Asensus Surgical, Durham, North Carolina, United States): The Senhance system stands out with its unique features, including haptic feedback and eye-tracking capabilities. These attributes provide surgeons with advanced control and visualisation during breast surgery procedures. Haptic feedback allows the surgeon to sense and control the robotic instruments more intuitively, leading to increased precision and a heightened sense of touch, which is vital in breast surgeries [9].

Versius® Surgical Robotic System (CMR Surgical, Cambridge, United Kingdom): The Versius system is celebrated for its compact design and portability, making it a favoured choice when limited workspace is a concern. In breast surgery, where delicate manoeuvres and confined anatomical structures are both important, the Versius system’s adaptability becomes advantageous. It allows surgeons to navigate tight spaces while maintaining the advantages of robotic assistance [10]. The Versius system strongly emphasises patient-centric care, primarily aiming to reduce trauma during breast surgery. It strives to minimise invasiveness and improve patient outcomes. Doing so aligns with the broader trend in healthcare towards more personalised and less invasive treatments, which can be particularly beneficial in the context of breast surgery, where preserving the patient’s well-being is paramount [11].

Comparison of Different Robotic Platforms

Surgical precision and dexterity: The ability of each robotic system to enhance surgical precision and dexterity during breast surgeries is paramount. This includes assessing how effectively the system aids in delicate tasks such as tissue removal, suturing, and dissection. An evaluation of the degree of control, instrument articulation, and tactile feedback provided by each system will be essential in determining its efficacy in achieving precise and intricate manoeuvres within the confines of the breast anatomy [12].

Imaging and visualisation: The quality of imaging and visualisation capabilities is pivotal, particularly in breast-conserving surgeries and nipple-sparing mastectomies. Superior imaging can significantly impact the surgeon’s ability to make informed decisions, identify critical structures, and navigate complex anatomical features. Precise, high-resolution imaging and effective visualisation systems can significantly enhance the accuracy and safety of breast surgery procedures [13].

Portability and ergonomics: A robotic system’s convenience and ergonomic design are crucial factors that influence the surgeon’s physical comfort and adaptability to different surgical settings. The system’s portability, including its size and ease of setup, is pivotal in seamlessly integrating into diverse operating environments. An ergonomic design enhances the surgeon’s comfort during lengthy procedures, reducing fatigue and the risk of musculoskeletal strain [14].

Adaptability to various breast surgery procedures: The extent to which each robotic system can be utilised in various breast surgery procedures is crucial. Breast surgery encompasses a spectrum of procedures, from mastectomies to lumpectomies and breast reconstructions. A versatile system that can adapt to different surgical contexts is highly desirable, as it allows for a comprehensive and consistent approach to breast surgery [15].

Advantages and limitations of using robotics in breast surgery

Advantages

Enhanced precision: One of the critical advantages of breast surgery robotic technology is its ability to offer enhanced precision. Robotic systems are designed to execute fine-tuned, delicate movements with high accuracy. This precision is particularly beneficial in tissue dissection, suturing, and tumour removal, where even the slightest deviation can have significant consequences. By reducing the risk of human error, robotics enhances patient safety and contributes to optimal surgical outcomes. Surgeons can control robotic instruments with utmost precision, minimising the potential for complications and promoting a successful recovery for breast surgery patients [16].

Improved visualisation: Integrating 3D visualisation and magnification in robotic breast surgery offers a significant advantage in surgical visualisation. This advanced imaging technology gives surgeons a clear and detailed view of the surgical site. This high-resolution view aids in identifying vital structures, such as blood vessels and nerves, and ensures the accurate delineation of tumour margins. The improved visualisation contributes to the procedure’s precision. It reduces the risk of unintentional damage to healthy tissue, which is crucial for preserving the patient’s overall health and post-surgery cosmetic results [17].

Reduced surgeon fatigue: Performing complex breast surgeries can be physically demanding for surgeons, particularly during lengthy procedures. Robotic systems can help alleviate this physical strain and reduce surgeon fatigue. These systems translate the surgeon’s hand movements into precise robotic actions, reducing the effort required to control instruments and perform intricate tasks. As a result, surgeons can maintain a higher level of concentration and dexterity throughout the surgery. Reduced surgeon fatigue enhances the procedure’s safety and allows surgeons to deliver high-quality care, even during extended surgeries, consistently. This aspect of robotic technology supports the sustainability of complex breast surgery practices over time [17].

Limitations

Cost: Acquiring and maintaining robotic systems is a significant drawback. These systems are often a substantial financial investment for healthcare facilities, and their ongoing maintenance and upgrades can be expensive. As a result, the initial financial barrier may limit the accessibility of robotic technology to smaller healthcare facilities with more constrained budgets. This cost consideration raises questions about equity in healthcare access and the ability of patients at different institutions to benefit from the advantages of robotic-assisted breast surgery [18].

Training requirements: The effective operation of robotic systems in breast surgery necessitates specialised training for surgeons and operating room staff. This training can be time consuming, requiring dedicated efforts to become proficient in using the technology. Surgeons must adapt to different skills and techniques, which may lengthen the learning curve. Furthermore, the entire surgical team must be well versed in the robotic procedures to ensure seamless coordination in the operating room. The need for extensive training can be a logistical challenge for healthcare institutions, affecting the integration of robotic technology into their surgical practices [19].

Limited haptic feedback: Some robotic systems are limited in providing the surgeon with haptic (tactile) feedback. Haptic feedback is crucial in traditional surgery, allowing surgeons to feel and respond to tissue characteristics such as texture, firmness, or resistance. The absence or limited nature of this feedback in robotic surgery can pose challenges, as it may affect a surgeon’s ability to assess tissue in real time. While visual and auditory cues are available, the absence of tactile feedback may lead to a perceived loss of the surgeon’s “touch,” potentially impacting decision-making and precision in certain aspects of the procedure [20].

Technical challenges: Robotic systems, like any technology, are susceptible to technical malfunctions and software glitches. These unexpected issues can disrupt surgery and require swift troubleshooting and resolution. The potential for technical challenges underscores the importance of having a well-trained support team and established protocols for handling such situations in the operating room. While robotic systems are designed to enhance surgical precision and safety, technical issues can introduce an element of unpredictability and stress into the surgical environment. Surgeons and staff must be prepared to address these challenges effectively to ensure patient safety and the procedure’s success [21].

Robotic applications in breast surgery

Preoperative Planning

Imaging and 3D modelling: Robotic systems are integrated with advanced imaging technologies, such as MRI and CT scans, to create precise 3D models of the patient’s breast. These models allow surgeons to accurately assess breast anatomy, tumour location, and blood supply. This information optimises surgical plans for minimal invasiveness, reduced complications, and improved cosmetic outcomes [22].

Surgical simulation: Before surgery, surgeons can use robotic systems for simulation and rehearsal. This involves practising the intended surgical procedure on the 3D models created during preoperative planning. Surgical simulation helps refine the surgical approach, familiarise the surgical team with the robotic system’s controls, and reduce the learning curve for complex procedures [23].

Intraoperative Procedures

Nipple-sparing mastectomy: In cases where a mastectomy is required, robotic systems have enhanced the feasibility of nipple-sparing mastectomies. By providing high-definition 3D visualisation and precise control, robots aid in preserving the nipple and areola, resulting in improved cosmetic outcomes and greater patient satisfaction. The robot’s dexterity allows for careful dissection of the breast tissue while avoiding damage to the nipple-areolar complex [24].

Lumpectomy and tumour resection: Robotics offer improved precision and minimal tissue damage for patients undergoing breast-conserving surgery. Surgeons can perform lumpectomies and tumour resections with high accuracy, minimising the removal of healthy tissue and ensuring clear margins around the tumour [15].

Sentinel lymph node biopsy: Sentinel lymph node biopsies are crucial for assessing the extent of cancer spread during breast cancer surgeries. Robotic systems facilitate the precise identification and removal of sentinel lymph nodes, reducing the risk of complications and postoperative lymphedema [25].

Postoperative Applications

Breast reconstruction: Following mastectomy or breast-conserving surgery, breast reconstruction can be vital to the patient’s journey. Robotic systems aid in autologous tissue flap procedures, such as deep inferior epigastric perforator flaps, to reconstruct the breast. Robotics enhance the precision and reliability of these complex microsurgical techniques [26].

Scar minimisation and aesthetic enhancement: Robotics can assist in suturing and tissue handling, leading to finer incision closure and scar minimisation. The precision of robotic suturing promotes improved cosmetic results, which is particularly significant in aesthetic breast procedures [27].

Patient outcomes and quality of life improvements

Cosmetic Outcomes

Robotic surgery is pivotal in enhancing cosmetic outcomes in breast surgery. The precision and dexterity of robotic instruments enable surgeons to perform intricate tasks with exceptional accuracy. This precision is particularly beneficial for procedures like breast reconstruction and augmentation, where preserving the nipple-areolar complex and minimising scarring are paramount. By facilitating precise tissue dissection, suturing, and implant placement, robotic technology allows for improved cosmetic results. Patients who undergo robotic-assisted breast surgery often experience less visible scarring, better symmetry, and a more natural appearance, which can significantly boost their confidence and satisfaction with the outcomes [15].

Psychological Well-Being

The cosmetic improvements from robotic breast surgery can profoundly impact patients’ psychological well-being. Reduced scarring and more aesthetically pleasing results can enhance a patient’s self-esteem and body image, potentially reducing feelings of self-consciousness and anxiety. The psychological benefits extend beyond physical appearance; patients may also experience improved emotional well-being, reduced stress, and greater body control. Ultimately, these positive psychological outcomes can contribute to a better quality of life for breast surgery patients in terms of their physical health and mental and emotional well-being [28].

Quality of Life

The impact of robotic-assisted breast surgery on the quality of life extends to various dimensions, including physical, emotional, and social aspects. Patients report reduced pain and discomfort and a quicker return to normal daily activities due to the minimally invasive nature of many robotic procedures. This improved physical well-being can lead to a higher overall quality of life as patients experience less disruption to their daily routines. Emotionally, the enhanced cosmetic outcomes and reduced complications associated with robotic surgery can increase self-confidence and create a more positive body image, promoting emotional well-being. Socially, patients may find it easier to engage in social and intimate relationships with renewed self-assurance. By examining patient-reported outcomes, we gain valuable insights into the comprehensive impact of robotic breast surgery on the overall quality of life, highlighting its benefits beyond the purely physical realm [29].

Complications and challenges in robotic breast surgery

Technical Challenges

Issues or complications can occasionally arise during robotic breast surgery. These may include system malfunctions, errors in the robotic platform, or limitations in the instruments used. These challenges can disrupt the surgical workflow and require rapid problem-solving. Surgeons and operating room staff must be well prepared to address technical issues and ensure the surgery proceeds smoothly. A failure to address these technical challenges effectively can directly impact patient safety and the procedure’s success [30].

Training and Learning Curve

Adopting robotic techniques in breast surgery presents a notable challenge for surgeons during the learning curve. Surgeons must invest time in acquiring the necessary skills and expertise to operate the robotic system effectively. Specialised training programs are often required to ensure competence. The learning curve can be associated with longer operating times and potentially higher risk of complications during the early stages of adoption. Therefore, institutions and surgeons must carefully plan to integrate robotic technology into their surgical practices, considering the training period and its potential effects on patient care [31].

Cost-Effectiveness

Evaluating the cost-effectiveness of robotic breast surgery compared to traditional methods is crucial regarding healthcare expenditure and resource allocation concerns. Robotic systems, their maintenance, and training programs can be costly. Assessing whether the benefits, such as improved outcomes and reduced complications, outweigh the expenses is vital for healthcare institutions and policymakers. Striking a balance between providing cutting-edge technology and managing healthcare costs is an ongoing challenge that must be addressed to ensure the responsible and sustainable adoption of robotic breast surgery [32].

Complications and Adverse Events

As with any surgical procedure, robotic breast surgery is not without potential complications and adverse events. These can include infections, bleeding, nerve damage, or anaesthesia-related complications. Careful monitoring and postoperative care are essential to promptly detect and manage these complications. It is imperative that surgeons and healthcare teams are well prepared to handle these potential issues, and patients should be informed about the possible risks during the informed consent process. Understanding these complications and adverse events is critical for the surgical team and the patient [33].

Patient Selection and Informed Consent

Proper patient selection for robotic breast surgery is crucial to ensure the procedure suits the individual’s condition and expectations. Patients must be assessed to determine if they are good candidates for robotic techniques. Additionally, obtaining informed consent is essential to ensure that patients have realistic expectations and fully understand the potential risks and benefits of the procedure. The informed consent process is critical to mitigating legal and ethical concerns and promoting patient autonomy and trust in the surgical team. Robotic surgeons and healthcare providers must prioritise patient education and engagement in decision-making to minimise potential challenges related to patient expectations and outcomes [34].

Surgeon training and learning curves

Training Programs for Robotic Breast Surgery

Structured training programs: Training programs are pivotal in preparing surgeons for robotic breast surgery. These programs are designed to provide a comprehensive understanding of the robotic system, its components, and the unique techniques required for robotic-assisted procedures. They typically encompass didactic education, classroom-style learning, and virtual simulation, allowing surgeons to practice their skills in a risk-free environment. Additionally, hands-on training with robotic platforms is a critical component, as it familiarises surgeons with the physical aspects of the system and its operation. These training programs ensure surgeons acquire the necessary skills and knowledge to safely and effectively perform robotic breast surgery [35].

Certification and credentialing: Certification and credentialing processes are established to validate a surgeon’s competence in operating robotic systems for breast surgery. These processes typically involve evaluating a surgeon’s training and experience and assessing their surgical skills using robotic platforms. By meeting specific criteria and demonstrating proficiency, surgeons can attain certification or credentials, recognising their ability to perform robotic-assisted breast surgeries. These processes are vital to upholding patient safety and ensuring that only qualified surgeons can use robotic technology in the operating room. They also contribute to standardising surgical skills in this field [36].

Continuing medical education: The field of robotic breast surgery, like all areas of medicine, is subject to continuous advancements and innovations. Ongoing education is essential to stay updated with the latest technology and surgical techniques. Continuing medical education (CME) programs and opportunities allow surgeons to expand their knowledge and skills. These programs may include workshops, conferences, and online courses, allowing surgeons to engage with experts and peers to share insights and experiences. Staying informed about the latest developments in robotic technology and breast surgery techniques is crucial for delivering the best possible patient care and maintaining the highest standards of practice. CME also reinforces the importance of lifelong learning in the medical profession [37].

Learning Curves for Surgeons Adopting Robotic Techniques

Early proficiency: Adopting robotic techniques in breast surgery typically involves an initial phase marked by a steep learning curve. Surgeons are transitioning from traditional surgical methods to robotic systems during this period. As they become familiar with the technology and its intricacies, they may experience longer surgical times and an increased risk of complications. Adjusting the robot’s interface, the precision required in instrument control, and adapting to a 3D visualisation system can be challenging. During this early phase, it is not uncommon for surgeons to seek guidance from mentors or proctors experienced in robotic surgery to navigate the learning curve effectively [38].

Skill acquisition: Learning the skills necessary for proficient robotic breast surgery is a gradual process. Surgeons must acquire proficiency in various aspects, including instrument control, hand-eye coordination, and adapting to 3D visualisation. Developing skill and precision with robotic instruments is a critical component of skill acquisition. Additionally, surgeons must become adept at interpreting the 3D visual information provided by the robotic system, which differs from the two-dimensional (2D) view in traditional surgery. This skill development often involves practice in virtual simulations and progressively complex surgical cases. As surgeons gain experience, they move through the learning curve, becoming more proficient and confident in robotic-assisted breast surgeries [39].

Case volume and complexity: The learning curve for adopting robotic techniques is influenced by the volume and complexity of cases a surgeon encounters. Surgeons progress from more straightforward to more complex cases as they gain experience. A higher case volume and variety contribute to skill development and increased proficiency. More challenging cases, such as breast reconstructions or procedures involving extensive tissue manipulation, provide valuable opportunities for surgeons to refine their techniques and decision-making. Confidence in robotic systems often grows as surgeons successfully manage various cases, making them more adaptable and capable of handling diverse patient needs. Ultimately, the learning curve gradually decreases as surgeons become more comfortable and efficient with robotic breast surgery [40].

Challenges and Strategies for Proficiency

Technical challenges: Surgeons face various technical challenges during the learning curve of adopting robotic techniques. These challenges may include instrument collisions, camera control to maintain the best view, and the need for precise hand movements to ensure accurate and safe surgical manoeuvres. Strategies to overcome these challenges often involve continuous practice, developing muscle memory, and gradually mastering the nuances of robotic system operation. Moreover, effectively troubleshooting technical issues is a valuable skill for surgeons during this phase [41].

Patient selection: Patient selection is crucial during the learning phase of robotic breast surgery. Surgeons should be cautious when choosing patients for these procedures, mainly as they build their proficiency. Opting for relatively straightforward and less complex cases can help mitigate risks and ensure optimal patient outcomes. Carefully assessing a patient’s suitability for robotic surgery is vital to balance the learning process with the patient’s safety and well-being [42].

Mentorship and proctoring: Mentorship and proctoring are vital in supporting surgeons during the learning curve. Experienced robotic breast surgeons can serve as mentors or proctors, guiding and overseeing the work of those in the learning phase. They provide valuable insights, share expertise, and offer real-time feedback to help the surgeon navigate challenges and build confidence. This direct mentorship can significantly expedite the learning process and enhance the safety and effectiveness of robotic breast surgeries [43].

Simulated training: Simulated training using robotic surgery simulators is an invaluable strategy for improving surgical skills and building confidence. These simulators replicate the conditions and challenges of actual robotic surgeries, allowing surgeons to practice without the pressure of the operating room. Simulated training enables surgeons to hone their skills, refine their technique, and become familiar with the robotic system’s interface and instruments. It is a safe and controlled environment to make and learn from mistakes, which is crucial for proficiency [44].

Team training: Proficiency in robotic breast surgery is not limited to surgeons alone; it extends to the entire surgical team. Nurses, surgical technicians, and assistants must also be trained to work seamlessly with the robotic system, understanding their roles and responsibilities during the procedure. Effective teamwork is crucial for the efficient operation of robotic systems in the operating room, and team training ensures that all surgical team members can collaborate effectively to achieve optimal outcomes [45].

Monitoring and evaluation: Ongoing monitoring and evaluation of a surgeon’s progress during the learning curve are essential to ensure patient safety and the quality of care. Regular assessment and feedback help identify areas that need improvement and allow for timely intervention. Surgical programs need to have a structured system for monitoring and evaluating a surgeon’s proficiency in robotic breast surgery, with clear criteria for progression and the ability to provide additional support and training as needed [46].

Patient experience and satisfaction

Patient Perspectives on Robotic Breast Surgery

Patient perspectives offer valuable insights into the world of robotic breast surgery. Real-life testimonials and personal accounts from those who have undergone this innovative approach provide a window into their experiences, emotions, and expectations before and after the procedure. These firsthand narratives shed light on the impact of robotic technology on the patient journey, allowing us to better understand the human aspect of these surgical advancements [5].

Furthermore, patient surveys and feedback play a crucial role in capturing the collective sentiment of individuals who have experienced robotic surgery. These surveys delve into various aspects of their experiences, ranging from their comfort level throughout the process to the effectiveness of communication with the surgical team, ultimately culminating in an assessment of their overall satisfaction. This data is instrumental in assessing and improving the patient-centred aspects of robotic breast surgery [47].

In addition, the role of shared decision-making between patients and healthcare providers in the context of robotic breast surgery is explored. This involves examining the information provided to patients regarding the benefits and risks associated with this innovative approach. Understanding the dynamics of this shared decision-making process is pivotal in ensuring that patients are well informed, actively engaged in their healthcare choices, and empowered to make decisions aligned with their individual needs and preferences [48].

Aesthetic and Functional Outcomes

Cosmetic outcomes: The aesthetic dimension of robotic breast surgery is pivotal, as it profoundly impacts a patient’s self-esteem and body image. This facet of the review delves into the meticulous assessment of how robotic techniques influence cosmetic outcomes. It explores explicitly preserving the delicate nipple-areolar complex, a highly sought-after goal in breast surgery. Robotic systems, with their unparalleled precision, facilitate the preservation of these vital structures, contributing to a remarkable improvement in the final appearance of the breast. Additionally, this section explores the implications of minimal scarring, a hallmark of robotic breast surgery, and how it contributes to the overall satisfaction and well-being of the patient. Furthermore, the pursuit of improved breast symmetry, a complex aspect of breast surgeries, is discussed, shedding light on how robotics plays a pivotal role in achieving harmonious and aesthetically pleasing results [49].

Functional outcomes: Beyond aesthetics, robotic breast surgery’s functional dimension is paramount. This review portion examines how robotic surgery influences functional aspects of patient well-being. It scrutinises how patients experience improved range of motion in their daily activities post-surgery. Additionally, this section explores the impact of robotic breast surgery on recovery time, including factors that expedite or facilitate the return to regular daily activities. Considering these functional aspects, this part of the review provides a holistic perspective on how robotic breast surgery enhances patients’ overall quality of life [50].

Long-term aesthetic and functional results: The evaluation of long-term aesthetic and functional outcomes in robotic breast surgery unfolds as a crucial aspect of understanding the sustained impact of these innovative techniques. Over time, the meticulous approach of robotic surgery manifests in enduring aesthetic improvements, with scarring evolving into refined, inconspicuous marks. The consideration of potential revision surgeries in the years following the initial procedure illuminates the comprehensive nature of patient care. Additionally, the sustained functional results come into focus, examining how patients continue to experience enhanced range of motion and shortened recovery times throughout their daily activities in the long term. The enduring positive outcomes affirm the role of robotic breast surgery in not only delivering immediate benefits but also in contributing to the sustained well-being and satisfaction of patients over an extended period. This emphasis on the durability of results underscores the transformative potential of robotic innovations in shaping the long-term trajectory of patients' lives post-surgery [51].

Psychological Impact and Satisfaction

Psychological well-being: Exploring the psychological well-being of individuals undergoing robotic breast surgery unveils a crucial dimension in the comprehensive assessment of patient outcomes. This facet delves into the intricate interplay between the innovative surgical approach and its impact on patients' mental and emotional health. Robotic breast surgery, with its focus on minimal scarring and enhanced cosmetic outcomes, emerges as a pivotal factor influencing psychological well-being. The reduction in visible scarring and the attainment of aesthetically pleasing results contribute to heightened self-esteem and body image. Patients often experience a positive shift in their emotional well-being, characterized by reduced stress and enhanced control over their body's appearance. The psychological benefits extend beyond the physical realm, addressing the emotional recovery of individuals. Factors such as body image, self-esteem, and overall emotional resilience undergo positive transformations, fostering a sense of empowerment and confidence. Understanding the psychological impact of robotic breast surgery underscores its potential to not only enhance the physical aspects of patient well-being but also contribute significantly to a positive and resilient mental health outlook. As patients navigate the transformative journey of breast surgery, the integration of robotic innovations emerges as a promising ally in fostering psychological well-being and holistic recovery [52].

Quality of life: Examining the impact of robotic breast surgery on the quality of life provides a comprehensive understanding of the broader dimensions that influence patients' well-being. The innovative techniques associated with robotic surgery contribute to a multifaceted enhancement in various aspects of patients' lives. The minimally invasive nature of many robotic procedures reduces postoperative pain and discomfort, facilitating a quicker return to normal daily activities. This improvement in physical well-being translates into a higher quality of life for patients, as they experience less disruption to their routines and a faster recovery. Beyond the physical realm, the positive cosmetic outcomes and reduced complications associated with robotic surgery are crucial to bolstering emotional well-being. Patients often report increased self-confidence, a positive body image, and reduced feelings of self-consciousness, contributing to an improved emotional quality of life. Moreover, the positive impact on social interactions and intimate relationships is noteworthy, as patients may find it easier to engage in social activities with renewed self-assurance. By exploring and understanding these dimensions, assessing quality of life becomes integral to recognizing the holistic benefits of robotic breast surgery for patients' lives. As we navigate the evolving landscape of breast surgery, the integration of robotic innovations emerges not only as a technological advancement but also as a transformative force, enhancing the overall quality of life for individuals undergoing these procedures [29].

Patient satisfaction: Patient satisfaction is a pivotal aspect in evaluating the success and impact of robotic breast surgery. This innovative approach to breast surgery, with its precision and advanced techniques, often correlates with heightened patient contentment. The meticulous use of robotic technology in procedures such as breast reconstruction and lumpectomy contributes to improved cosmetic outcomes, including minimal scarring and enhanced symmetry. Such aesthetic enhancements positively influence patient confidence and body image, aligning postoperative results closely with individual expectations. Additionally, the reduced postoperative pain and quicker recovery associated with many robotic procedures contribute to an overall positive patient experience. The alignment of outcomes with patient expectations, effective pain management, and a smoother recovery process collectively contribute to increased satisfaction. Patient satisfaction extends beyond the physical aspects to encompass the emotional and psychological dimensions. By exploring patient-reported outcomes and feedback, we gain valuable insights into the nuanced elements contributing to overall satisfaction, including communication with the surgical team and comfort levels throughout the process. As we delve into patient satisfaction with robotic breast surgery, it becomes evident that integrating advanced robotic technologies not only advances surgical techniques but also significantly enhances the overall experience and contentment of individuals undergoing these transformative procedures [53].

Future directions and innovations

Emerging Technologies in Robotic Breast Surgery

Enhanced imaging and navigation: Incorporating enhanced imaging and navigation technologies stands at the forefront of transformative advancements in robotic breast surgery. These innovations, such as real-time MRI and intraoperative ultrasound, revolutionize the precision and decision-making processes during surgical procedures. Real-time MRI capabilities offer continuous, high-resolution surgical site visualization, providing invaluable insights for procedures like lumpectomies and tumor resections. In parallel, integrating intraoperative ultrasound facilitates real-time assessment of tissue characteristics, guiding surgeons in making informed decisions and ensuring precise navigation. These technologies serve as a navigational compass, empowering surgeons to optimize incision placement, tumor resection boundaries, and overall procedural strategy. The impact of these enhanced imaging and navigation tools extends to tangible patient benefits, including minimal tissue damage, reduced surgical time, and increased accuracy in tumor removal. As a result, patients experience improved cosmetic outcomes, diminished postoperative complications, and accelerated recovery, enhancing their overall quality of life. The ongoing evolution of these technologies holds great promise for further elevating the efficacy and outcomes of robotic breast surgery, marking a significant milestone in the pursuit of precision and safety in breast surgical interventions [54].

Miniaturisation and portability: The development of smaller and more portable robotic systems marks a transformative shift in the field of breast surgery, and this section examines the implications of this innovation. It explores how the trend towards miniaturisation and increased portability is expanding the use of robotic systems in various clinical settings. These compact robotic platforms allow surgeons to utilise robotics in settings previously constrained by space or resource limitations. This development has the potential to enhance access to advanced surgical techniques, ultimately benefitting patients who may require breast surgery in diverse healthcare environments [55].

Intraoperative sensors: Integrating sensors into robotic breast surgery is a promising development. Sensors can provide real-time feedback on tissue characteristics, blood flow, and tissue viability during surgery. These sensors offer crucial data to surgeons, aiding in decision-making by providing insights into the health of the tissues being manipulated. The review outlines how this technology can improve surgical precision and safety, allowing surgeons to adapt their techniques in response to real-time information. By incorporating these sensors, robotic breast surgery can become even more patient centred and outcome driven, making decisions based on the conditions encountered during surgery [56].

Integration of Artificial Intelligence (AI) and Machine Learning

Image analysis and tumour detection: AI algorithms’ deployment in breast surgery transforms how tumours are detected and characterised from medical images. This section underscores how AI assists in the automated detection and classification of breast tumours, contributing to improved diagnostic accuracy and reduced false positives. By efficiently processing and analysing vast quantities of medical images, AI empowers radiologists and clinicians to make more precise and timely decisions, enhancing patient outcomes and minimising unnecessary interventions [57].

Predictive analytics: Machine learning models can revolutionise surgical planning and patient care. Machine learning algorithms can be harnessed to predict surgical outcomes, complications, and patient responses. Machine learning models can generate personalised patient treatment plans by leveraging vast datasets and considering numerous variables. This personalisation enhances patient outcomes, reduces the risk of complications, and accelerates recovery, making breast surgery more patient centred [58].

Surgical assistance: The development of AI-powered robotic surgical assistants signifies a significant leap forward in pursuing precision and safety in breast surgery. These intelligent robotic assistants can work synergistically with human surgeons, enhancing the precision and safety of surgical procedures. By leveraging real-time data analysis and decision-making, these AI-powered assistants support surgeons, ensuring that each procedure step is carried out accurately and carefully. This collaborative approach promises to improve surgical outcomes and minimise complications, ultimately redefining the standard of care in breast surgery [59].

Potential Advancements in Telesurgery and Remote Assistance

Telesurgery: The future of telesurgery is an exciting frontier that promises to improve access to specialised care. In telesurgery, surgeons can remotely perform surgeries on patients in different locations and this innovative approach has the potential to bridge the gap between patients in underserved or remote areas and highly skilled surgeons. Telesurgery promises to provide timely access to expert care, especially in cases that demand specialised knowledge and skills, ultimately enhancing the overall reach of robotic breast surgery and increasing the accessibility of high-quality surgical procedures [60].

Remote assistance: Using robotics for remote assistance and consultation represents a significant leap forward in the support structure of breast surgery. This section delves into how experienced surgeons can remotely guide and assist less experienced surgeons during procedures in real-time. It outlines the potential for leveraging the precision of robotic systems to provide critical assistance, especially in complex and challenging cases. This approach enhances the collaborative nature of surgical care. It contributes to the development of less experienced surgeons while ensuring that patients receive the best possible care, regardless of their location [61].

Training and education: The potential for remote robotic surgery training and education programs is an educational paradigm shift. It explores how surgeons can acquire proficiency in robotic breast surgery without geographical limitations, thanks to remote training and educational platforms. This section underscores the importance of disseminating knowledge and expertise in robotic breast surgery and provides a pathway for the next generation of surgeons to attain proficiency. By transcending geographical constraints, remote training and education programs enable a wider pool of healthcare professionals to harness the benefits of robotics and provide advanced surgical care to patients [62].

## Conclusions

The integration of robotic innovations into breast surgery marks a transformative phase in surgical techniques. This comprehensive review has highlighted the advantages of robotic systems, including enhanced precision, improved visualisation, and reduced surgeon fatigue. The varied applications, from preoperative planning to postoperative outcomes, underscore the technology’s impact on cosmetic results and patients’ psychological well-being. Despite the promising advancements, challenges such as cost considerations, training requirements, and potential complications require ongoing attention. Looking ahead, continued collaboration and research are crucial for unlocking the full potential of robotic-assisted breast surgery. The journey has just begun, and with sustained commitment, we anticipate a future of safer, more accessible, and more effective breast surgery, ultimately enhancing the lives of those facing breast-related health challenges.
